# Synthesis of an organic-soluble π-conjugated [3]rotaxane via rotation of glucopyranose units in permethylated β-cyclodextrin

**DOI:** 10.3762/bjoc.10.297

**Published:** 2014-11-28

**Authors:** Jun Terao, Yohei Konoshima, Akitoshi Matono, Hiroshi Masai, Tetsuaki Fujihara, Yasushi Tsuji

**Affiliations:** 1 Department of Energy and Hydrocarbon Chemistry, Graduate School of Engineering, Kyoto University, Nishikyo-ku, Kyoto, Japan

**Keywords:** cross-coupling reaction, insulated π-conjugated molecule, oligothiophene, permethylated cyclodextrin, [3]rotaxane

## Abstract

We synthesized symmetrically insulated oligo(*para*-phenylene) and oligothiophene with a pseudo-linked [3]rotaxane structure by full rotation of glucopyranose units via a flipping (tumbling) mechanism in the π-conjugated guest having two permethylated β-cyclodextrin units at both ends. We also succeeded in the synthesis of an organic-soluble fixed [3]rotaxane by a cross-coupling or complexation reaction of thus formed pseudo linked [3]rotaxane. Oligo(*para*-phenylene), oligothiophene, and porphyrin derivatives were used as π-conjugated guests with stopper groups.

## Introduction

**Figure 1:**
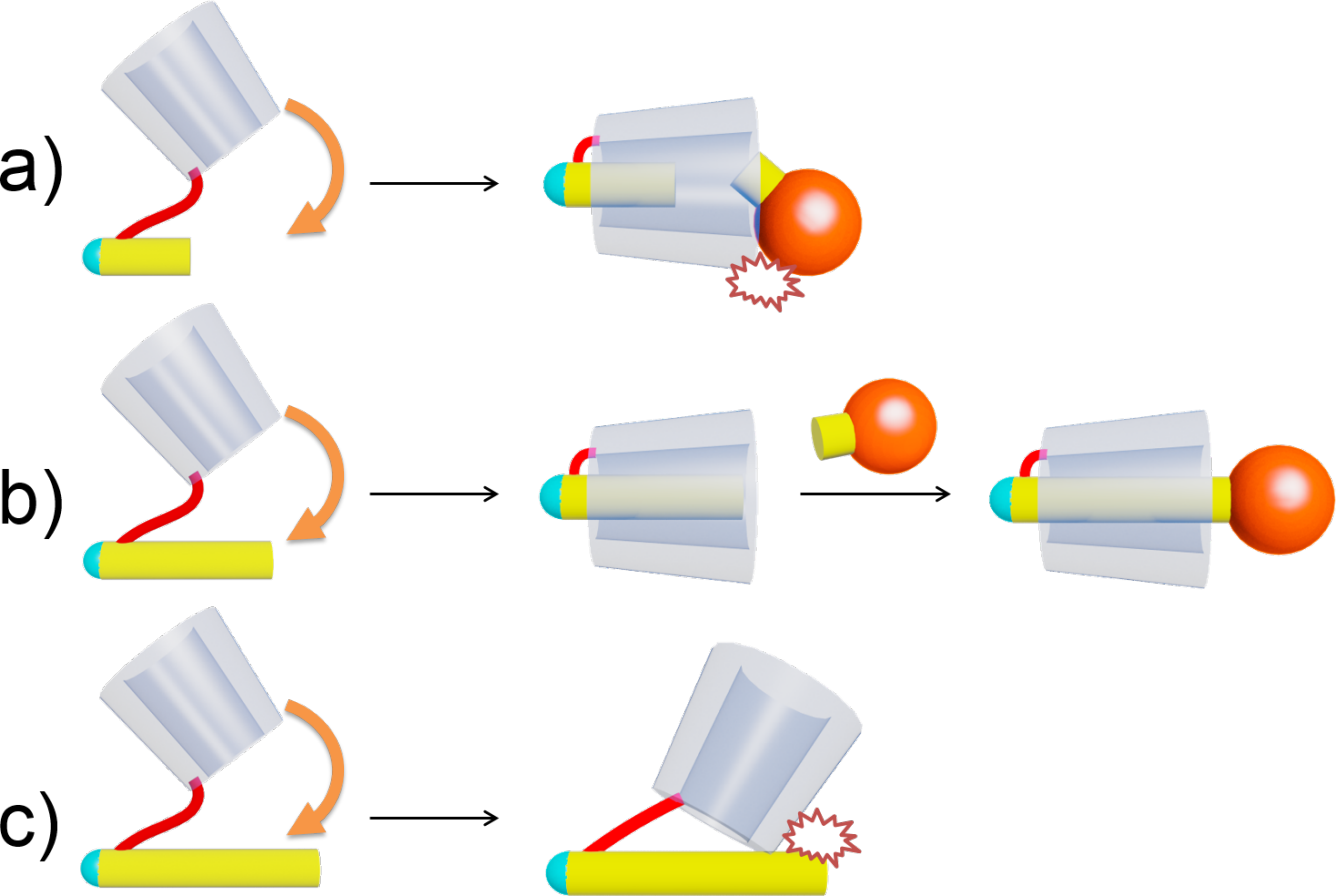
Synthesis of [1]rotaxane by self-inclusion of a host–guest-linked molecule: a) short molecular length, b) appropriate molecular length, c) long molecular length.

**Figure 2:**
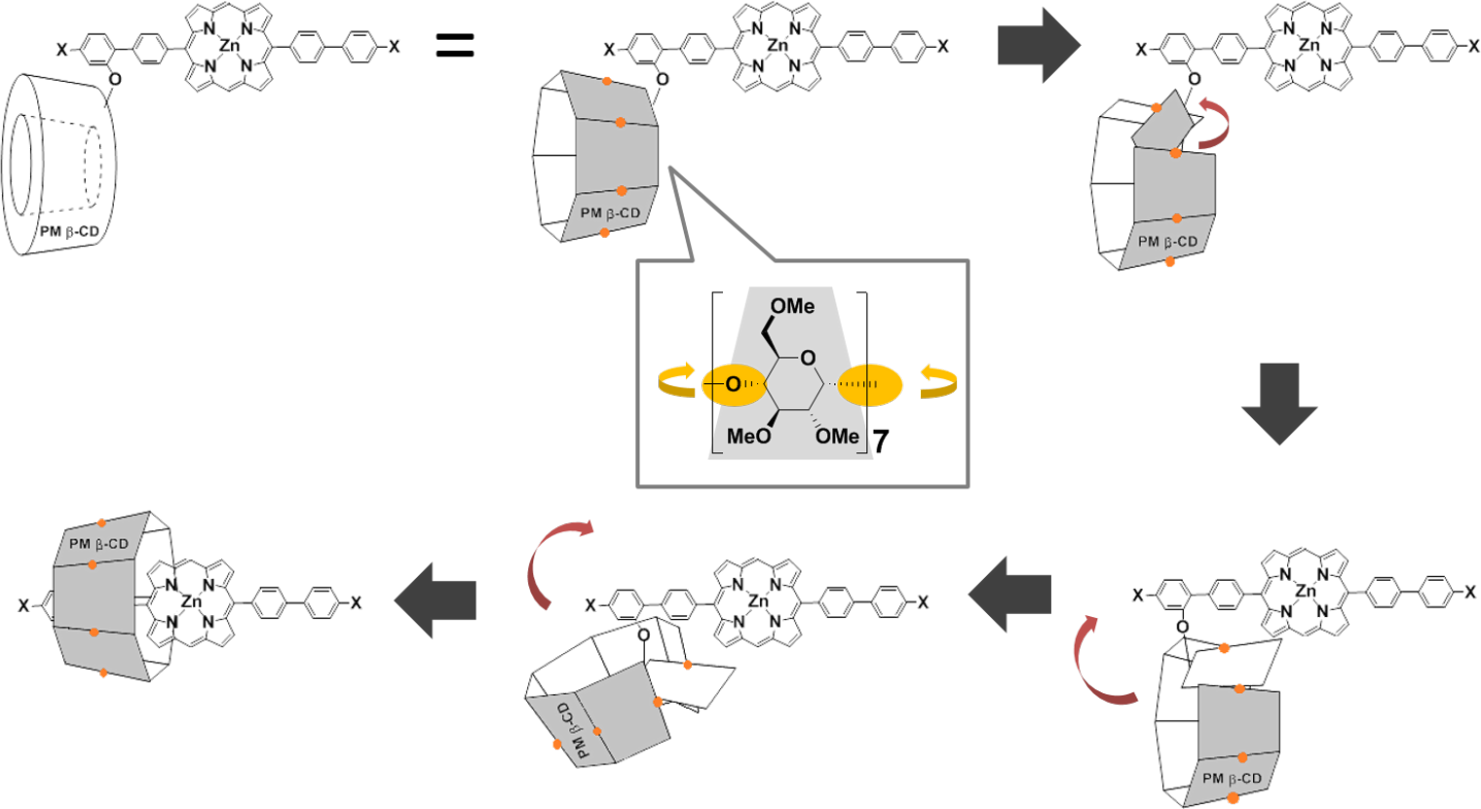
Synthesis of an insulated molecule via flipping phenomenon.

Insulated molecular wires (IMWs) [1–2]_,_ which feature π-conjugated polymer chains covered by protective sheaths, have attracted considerable attention as next-generation mono-molecular electronic devices because of their potential conductivity and luminescent properties [3–5]. Cyclodextrin (CD) derivatives are widely used as a protective sheath for the synthesis of IMWs because they are easily obtainable and efficient in the inclusion of various polymers into their cavity via hydrophobic interactions in water [6–7]. General methods for the synthesis of π-conjugated polymer-based IMWs using CD derivatives involve (I) threading π-conjugated polymers through CD [8–9] using a method developed by Harada for the synthesis of a polyrotaxane [10], (II) polymerization of pseudo [2]rotaxane monomer formed by self-inclusion of a π-conjugated guest with CD [11], and (III) copolymerization of the thus-formed pseudorotaxane with linker molecules [12]. These polymers are soluble in water but generally insoluble in organic solvents; this is because the hydrophilic CD covers the organic soluble π-conjugated polymer chains [13]. Uncovered sites are also randomly present because of shuttling of CDs along the π-conjugated chain and remnant water molecules; these affect the charge-transport ability and are disadvantageous for the use of these IMWs as electronic materials. Therefore, we decided to use permethylated cyclodextrin (PMCD) provided by permethylation of all the hydroxy groups of CD. However, the low solubility of PMCD in water as compared with native CDs or other randomly methylated CDs such as 2,6-dimethyl-β-cyclodextrin impedes the formation of self-inclusion complexes via hydrophobic interactions in water. To rise above this problem, we prepared organic-soluble host–guest-linked PMCD derivatives that can undergo intramolecular self-inclusion to form an insulated molecule with an [1]rotaxane structure in methanolic aqueous solutions, where PMCD derivatives are soluble in. We recently developed a new method for synthesizing π-conjugated IMWs with polyrotaxane structures via polymerization of π-conjugated [1]rotaxane monomers bearing PMCDs as macrocycles [14]. Further, we confirmed that such IMWs with poly[1]rotaxane structure are highly soluble in a variety of organic solvents and have good rigidity, photoluminescence efficiency [15], and charge mobility [16–17]. A key step for the synthesis of these IMWs is the preparation of insulated π-conjugated monomers with [1]rotaxane structures by self-inclusion of π-conjugated monomer-linked PMCDs followed by elongation of the π-conjugated units via cross-coupling in a hydrophilic solvent, such as an aqueous 50% methanol solution [18]. For this method, it is necessary that the molecular length of the π-conjugated guest is less than the internal diameter of the CD and similar to the depth of PMCD in order to form the self-inclusion complex (Figure 1b). When the π-conjugated guest is shorter than the depth of PMCD, the cross-coupling reaction is strongly inhibited because the reaction has to occur in the PMCD cavity (Figure 1a). On the other hand, a self-inclusion complex does not form if the guest is longer than the internal diameter of PMCD (Figure 1c). Thus far, we have succeeded in the synthesis of insulated π-conjugated monomers with [1]rotaxane structures using oligo(phenylene–ethylene) units, which have the appropriate length, as the π-conjugated guests for the fixation of self-inclusion complexes by elongation of the guest unit via cross-coupling (Figure 1b). However, this method can only be applied to the synthesis of insulated oligo(phenylene–ethylene) monomers. To synthesize other insulated π-conjugated molecules, we became interested in another insulation technique to replace this self-inclusion: full rotation of the glucopyranose units of methylated cyclodextrin by alteration of the relative orientation of the D-glucopyranoside rings via a flipping (tumbling) mechanism as shown in Figure 2 [19–22]. From the results of our study on the synthesis of organic-soluble π-conjugated rotaxanes, we report herein the synthesis of insulated oligo(*para*-phenylene) [23] and oligothiophene [24] with linked [3]rotaxane structures via the flipping phenomenon [25].

## Results and Discussion

**Scheme 1:**
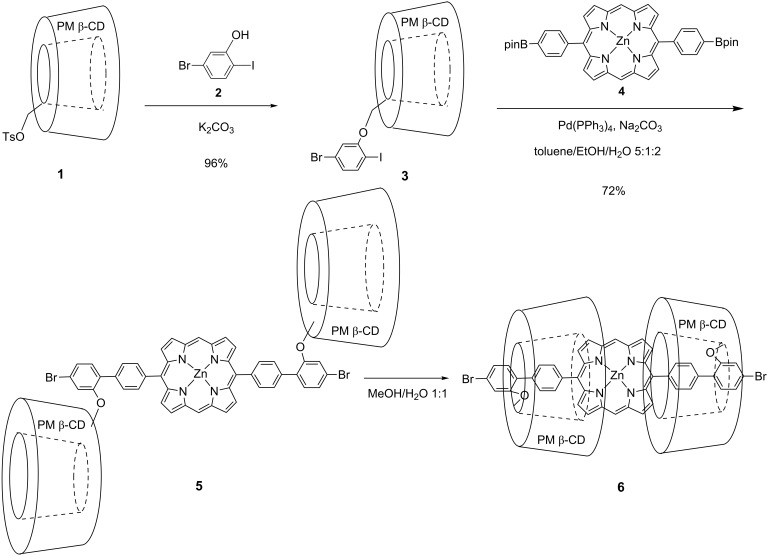
The synthetic route to the PMβ-CD based linked [3]rotaxane with a 5,15-di([1,1'-biphenyl]-4-yl)porphyrin backbone.

Kano and co-workers reported the synthesis of a linked pseudo [3]rotaxane involving a water-soluble porphyrin unit with carboxylate groups as the guest unit by double self-inclusion via sequential flipping of permethylated β-cyclodextrin (PM β-CD) in water [26]. To synthesize an organic-soluble insulated π-conjugated rotaxane, we applied this technique to the synthesis of an insulated porphyrin without water-soluble functional groups. The synthetic route to the PM β-CD based linked [3]rotaxane precursor with a 5,15-di([1,1'-biphenyl]-4-yl)porphyrin backbone as the guest unit is shown in Scheme 1. In order to fix the pseudo [3]rotaxane structure, we introduced two bromo groups (cross-coupling reaction point) at both terminal positions of the 5,15-di([1,1'-biphenyl]-4-yl)porphyrin unit and two PM β-CD groups at *meta* position to the bromo groups. 7-*O*-Monotosyl PM β-CD **1** was synthesized from a native β-cyclodextrin in two steps using an established protocol [27]. Reaction of **1** with 5-bromo-2-iodophenol (**2**) afforded benzene-linked PM β-CD **3**. The 2:1 Suzuki cross-coupling reaction of thus-formed **3** with dipinacolborane porphyrin derivative **4** gave rise to precursor of pseudo [3]rotaxane **5**. It should be noted that pseudo-linked [3]rotaxane **6** formed quantitatively via double self-inclusion through flipping of **5** in an aqueous 50% methanol solution.

**Figure 3:**
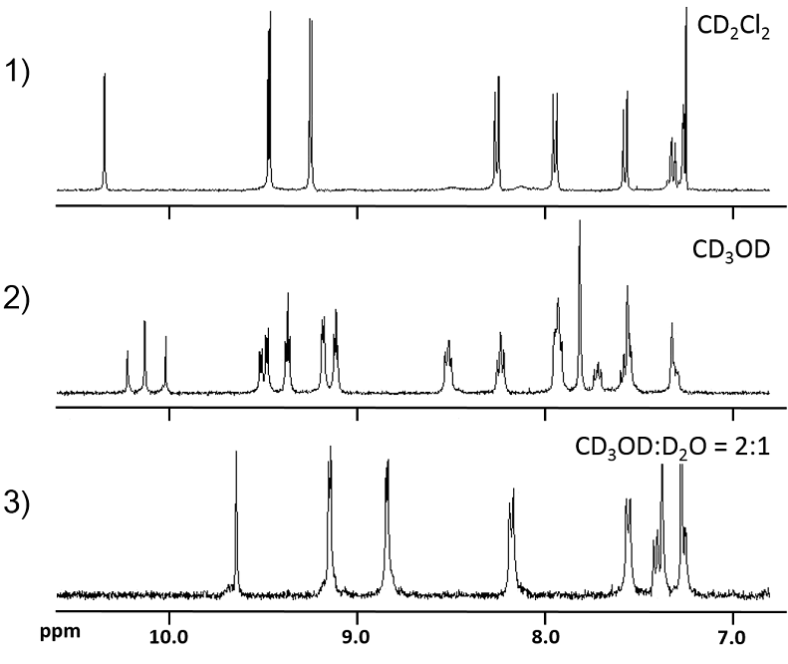
The aromatic region of the ^1^H NMR spectra of **5** at 25 °C: 1) CDCl_3_, 2) CD_3_OD, and 3) CD_3_OD:D_2_O 1:1.

This unique phenomenon has been characterized by ^1^H NMR in different solvents (Figure 3). The ^1^H NMR spectrum in CD_2_Cl_2_ reveals that the 5,15-di([1,1'-biphenyl]-4-yl)porphyrin moiety is excluded from the cavity of the PM β-CD, while the ^1^H NMR spectrum in CD_3_OD reveals a mixture of exclusion and inclusion complexes. On increasing the hydrophilicity of the solvent, the formation of double inclusion complex **6** predominated. When we added D_2_O in order to increase hydrophilicity, the exclusion complex completely converted into inclusion complex **6** in CD_3_OD:D_2_O = 1:1 solutions.

**Scheme 2:**
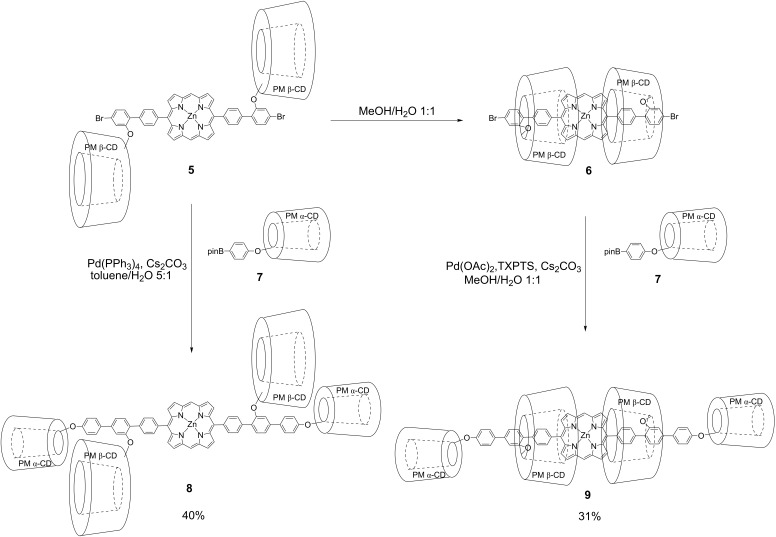
Selective synthesis of fixed [3]rotaxane by Suzuki cross-coupling reaction.

To synthesize an organic-soluble linked [3]rotaxane, we then fixed this pseudo rotaxane structure, which was only present in 50% methanol aqueous solution. The capping reaction by Suzuki cross-coupling with pinacolboron derivative **7** bearing PM α-CD as the bulky stopper group was carried out under the same hydrophilic solvent conditions as in the formation of **6** (Scheme 2). The desired fixed [3]rotaxane **9** was isolated in 31% yield by preparative size exclusion chromatography using CHCl_3_ as the eluent. The corresponding uninsulated compound **8** as a reference was also intentionally synthesized by the reaction of **5** with **7** in hydrophobic solvent (toluene/H_2_O 5:1) instead of hydrophilic solvent (CH_3_OH/H_2_O 1:1). Although the MALDI–TOF mass spectrum of **8** and **9** exhibited the same signal at *m*/*z* = 6097 corresponding to [**8** or **9** + Na]^+^, each ^1^H NMR spectrum of those showed the pure single product but completely different, respectively. These results suggest that we succeeded in the selective synthesis of **8** and **9** by simply changing the reaction solvent.

**Scheme 3:**
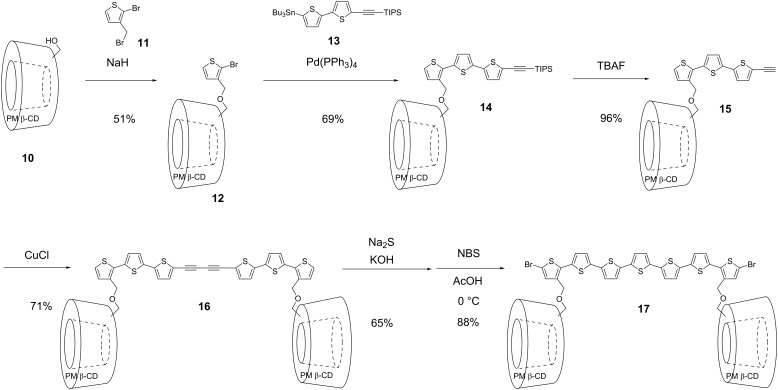
The synthetic routes to precursor of PM β-CD based insulated oligothiophene.

We next applied this synthetic method to other π-conjugated guests. Scheme 3 shows the synthetic routes to precursor of PM β-CD based insulated oligothiophene. Mono-6-desmethyl PM β-CD alcohol **10** was synthesized from **1** using sodium naphthalenide [27]. The reaction of the thus-formed monoalcohol with 2-bromo-3-(bromomethyl)thiophene (**11**) afforded thiophene-linked PM β-CD **12**. Pd-catalyzed Stille cross-coupling of **12** with bithiophene **13** bearing a silyl-protected alkynyl group afforded trithiophene-linked PM β-CD **14**. After the deprotection of the triisopropyl group of **14** and the Glaser dimerization reaction of **15**, symmetrical oligothiophene **16** bearing two PM β-CDs was obtained. Treatment of **16** with Na_2_S and KOH followed by dibromination by *N*-bromosuccinimide formed dibromo-heptathiophene **17** with two PMCDs.

**Scheme 4:**
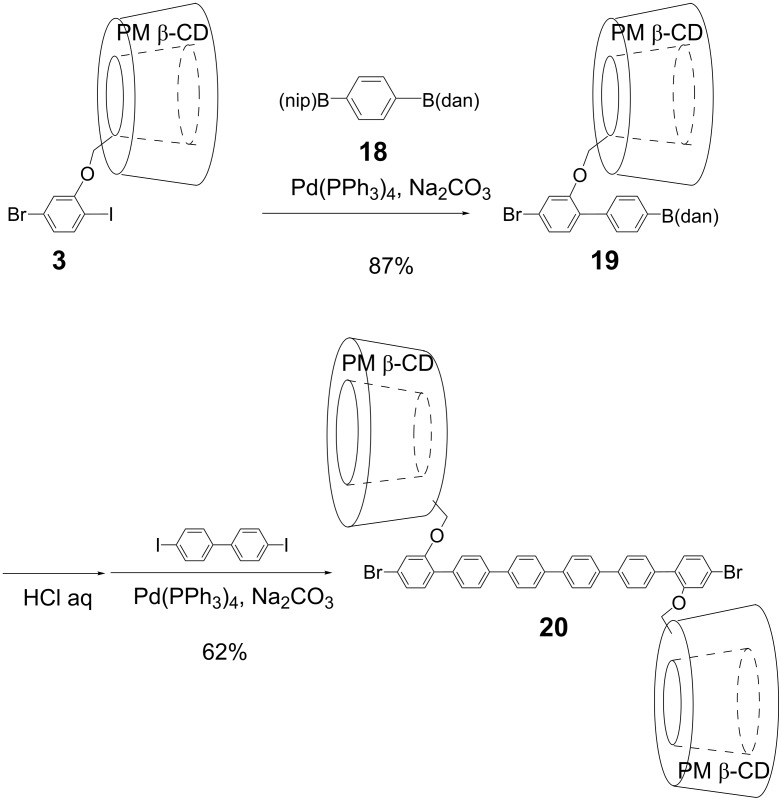
Synthesis of dibromohexa(*para*-phenylene) with two PMCDs **20**.

Scheme 4 shows the synthetic route to the precursor of PM β-CD-based insulated oligo(*para*-phenylene) **20**. Sequential Suzuki cross-coupling of **3** with unsymmetrically protected benzene-1,4-diboronic acid derivative **18** and diiodobiphenyl gave rise to hexa(*para*-phenylene) derivative **20**.

**Scheme 5:**
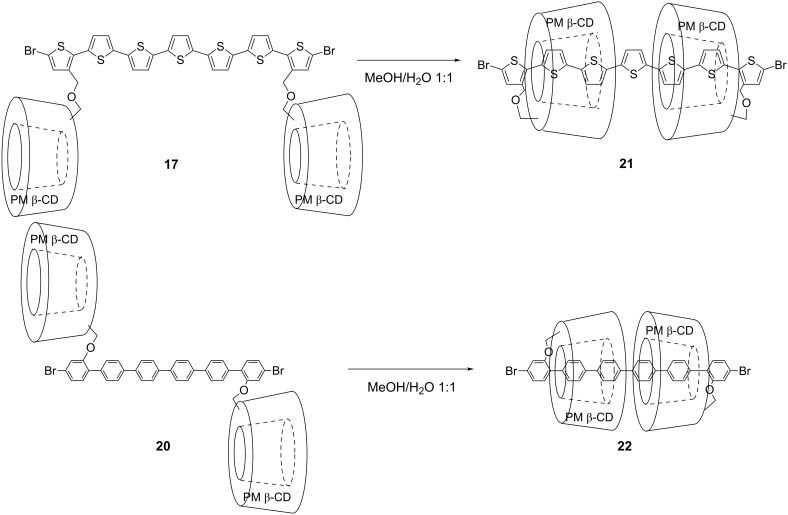
Synthesis of pseudo-linked [3]rotaxanes via double self-inclusion through flipping.

**Figure 4:**
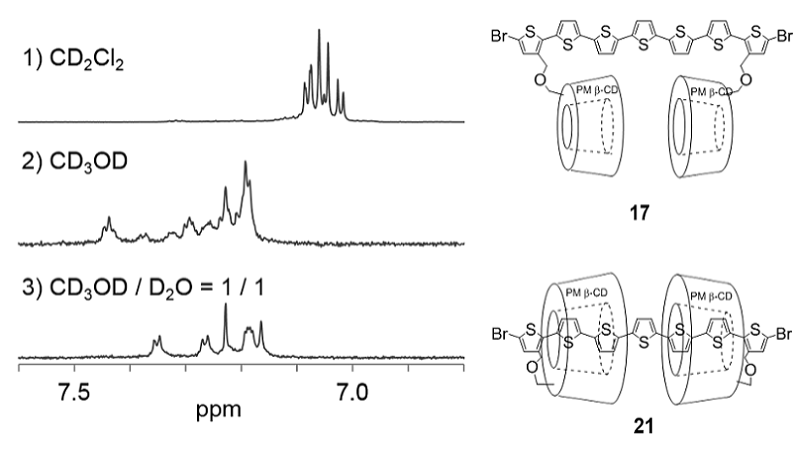
The aromatic region of the ^1^H NMR spectra of **5** at 25 °C: 1) CDCl_3_, 2) CD_3_OD, and CD_3_OD/D_2_O 1:1.

**Figure 5:**
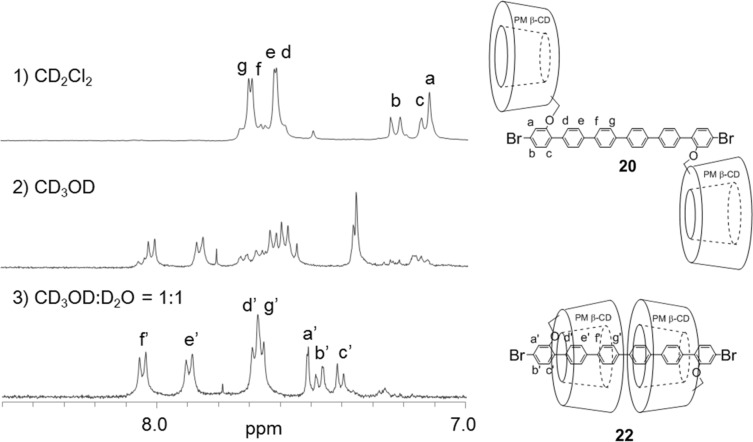
The aromatic region of the ^1^H NMR spectra of **20** at 25 °C: 1) CDCl_3_, 2) CD_3_OD, and CD_3_OD/D_2_O 1:1.

Pseudo linked [3]rotaxanes **21** and **22** were also formed quantitatively in an aqueous 50% methanol solution via double self-inclusion through flipping of **17** and **20**, respectively (Scheme 5). The formation of these inclusion complexes were also confirmed by ^1^H NMR spectroscopy in different solvents (Figure 4 and Figure 5, Supporting Information File 1, Figure S2). These linked [3]rotaxanes can be key monomers for synthesizing organic soluble IMWs bearing polythiophene or poly(*para*-phenylene) as backbone units.

**Scheme 6:**
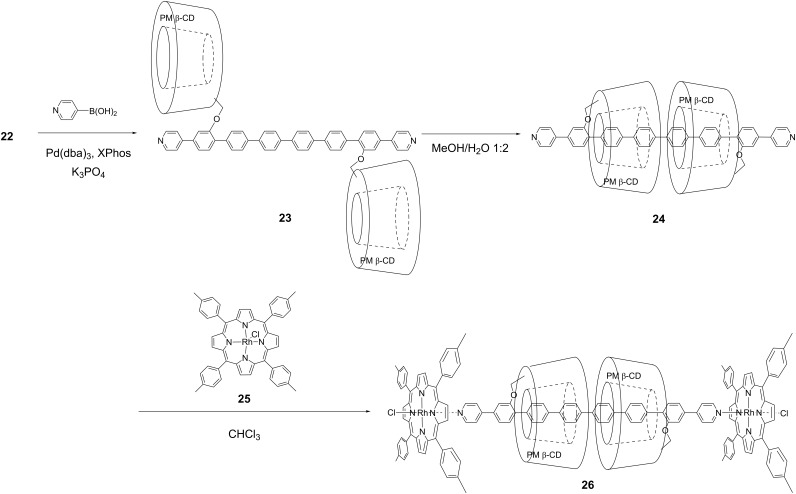
Synthesis of fixed [3]rotaxane via complexation with rhodium porphyrin.

We next fixed the pseudo rotaxane structure by complexation instead of cross-coupling reaction (Scheme 6). First, we introduced pyridyl groups at the terminal positions of hexa(*para*-phenylene) precursor **20** by Suzuki cross-coupling with *para*-pyridylboronic acid to generate **23**. After confirmation of the formation of pseudo [3]rotaxane **24** via flipping of **23** in CD_3_OD/D_2_O 1:2 solution by ^1^H NMR analysis, we capped this pseudo [3]rotaxane structure by reacting **24** with rhodium porphyrin complex **25** in chloroform to form fixed [3]rotaxane **26** in 17% isolated yield [28]. The structure of **26** was confirmed by ^1^H NMR analyses in CDCl_3_. The results suggest that **26** maintains a [3]rotaxane structure even in organic solvents because it is capped with sterically bulky porphyrin units, which impede the reverse flipping of the PM β-CD units.

**Figure 6:**
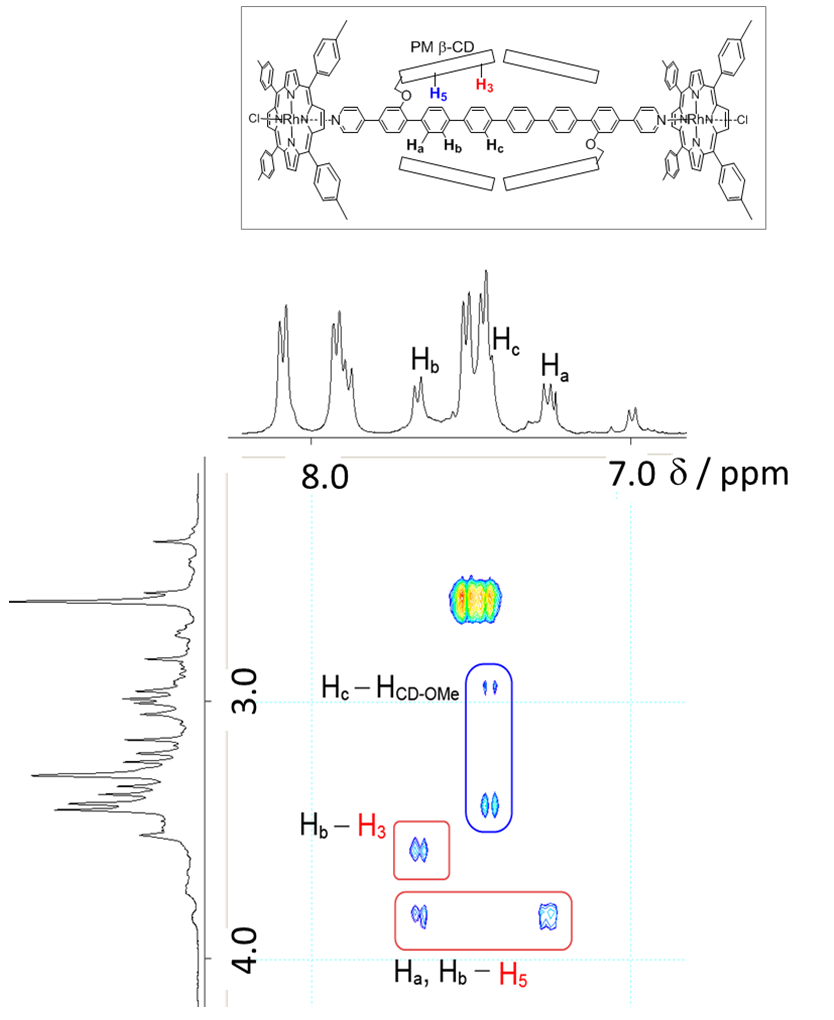
Partial ROESY NMR spectrum of **26** (400 MHz, CDCl_3_) showing the NOEs between aromatic protons of the axial hexa(*para*-phenylene) and inner protons of cyclodextrins.

As shown in Figure 6, the unique fixed [3]rotaxane structure of **26** was confirmed by 2D ^1^H ROESY NMR analysis: there are obvious nuclear Overhauser enhancements (NOEs) between the protons on the hexa(*para*-phenylene) unit and the H3 and H5 protons on the interior of the permethylated cyclodextrin [29]. These experimental results indicated that the rotaxane structure was constructed between PM β-CDs and *para*-phenylene as hosts and guest, respectively.

## Conclusion

We developed the synthesis of symmetrically insulated π-conjugated molecules with pseudo linked [3]rotaxane structure via flipping of a glucopyranose unit of permethylated β-cyclodextrin. In this method, oligo(*para*-phenylene), oligothiophene, and porphyrin derivatives were used as π-conjugated guests. After cross-coupling or complexation of the pseudo linked [3]rotaxane with capping molecules, the fixed [3]rotaxane was isolated in chloroform solution. The formation of the fixed [3]rotaxane was confirmed by NMR analysis. Experiments are now in progress towards synthesizing IMWs bearing polythiophene or poly(*para*-phenylene) as backbone units by polymerization of these linked [3]rotaxanes as monomers.

## Supporting Information

File 1 Experimental and analytical data.
