# Supplementary material for: Synthesis of an organic-soluble π-conjugated [3]rotaxane via rotation of glucopyranose units in permethylated β-cyclodextrin
Source: Beilstein J Org Chem. 2014 Nov 28;10:2800–8. doi: 10.3762/bjoc.10.297 (PMC4273295; doi:10.3762/bjoc.10.297)
Supplement: File 1 — Experimental and analytical data. [file Beilstein_J_Org_Chem-10-2800-s001.pdf]

Supporting Information

for

Synthesis of an organic-soluble π-conjugated [3]rotaxane via rotation of glucopyranose units in permethylated β-cyclodextrin

Jun Terao*, Yohei Konoshima, Akitoshi Matono, Hiroshi Masai, Tetsuaki Fujihara, and Yasushi Tsuji

Address: Department of Energy and Hydrocarbon Chemistry, Graduate School of Engineering, Kyoto University, Nishikyo-ku, Kyoto, Japan

Email: Jun Terao - terao@scl.kyoto-u.ac.jp

* Corresponding author

Experimental and analytical data

1.1 Materials

Unless otherwise noted, commercially available chemicals were used as received. Solvents were purified as follows: Reaction solvents were degassed through freeze-pump-thaw or argon bubbling before using. Dry toluene and THF were purchased from Kanto Chemical and further purified by passage through activated alumina under positive argon pressure as described by Grubbs et al.

1.2. Experimental

NMR Spectroscopy: ^{1}H NMR (400 MHz), ^{13}C {^{1}H} NMR (100 MHz) and ROESY NMR spectra were measured with a JEOL ECX-400 spectrometer and ^{1}H NMR (500 MHz), ^{13}C {^{1}H} NMR (126 MHz) were measured with a BRUKER AVANCE III HD 500 spectrometer. The ^{1}H NMR chemical shifts are reported relative to tetramethylsilane (TMS, 0.00 ppm) or residual protiated solvent in $CDCl_3$ (7.26 ppm) and in CD_2Cl_2 (5.32 ppm). The ^{13}C NMR chemical shifts are reported relative to tetramethylsilane (TMS, 0.00 ppm) or $^{13}CDCl_3$ (77.0 ppm).

Mass Spectrometry (MS): Matrix assisted laser desorption/ionization time-of-flight (MALDI-TOF) mass spectra were obtained with 1,8-dihydroxy-9(10H)-anthracenone (DIT) or α-cyano-4-hydroxycinnamic acid (CHCA) as a matrix and NaTFA as a cationization reagent on a SHIMADZU KRATOS TOF MASS spectrometer AXIMA-CFR Plus. High-resolution mass spectra (EI-HRMS and ESI-HRMS) were obtained with JEOL JMX-SX102A and Thermo SCIENTIFIC Exactive LC-MS spectrometers.

Silica gel Column chromatography: Column chromatography was carried out on silica gel (Kanto N60, spherical, neutral, 63-210 μm) or silica gel (Biotage, HP-Sphere or HP-SIL, neutral, 20 or 50 μm).

Preparative recycling gel permeation chromatography (GPC): Preparative recycling GPC was performed with a JAI LC9140 System equipped with JAIGEL-1H, 2H, 2.5H, and -3H columns, a JAI UV DETECTOR 310, and a JAI RI DETRCTOR RI-5, or performed with a

JAI LC−9130NEXT System equipped with JAIGEL−2H and −2.5H columns, a JAI UV−370 NEXT, and a JAI RI−700 NEXT, or performed with a SHIMADZU LC-20AP System equipped with a Shodex K-4003L column, a SHIMADZU SPD-20A, and a SHIMADZU RID-10A using $CHCl_3$ as the eluent at a flow rate of 14 mL min^{-1}.

Analytical size-exclusion chromatography (SEC): Analytical SEC was performed with a GL-Science GL-7400 HPLC System equipped with Shodex KF-801, -802, -802.5, -803, -804 columns, a GL-7410 HPLC pump, a GL-7400 UV detector, and a GL-7454 RI detector using THF as the eluent at a flow rate of 0.6 mL min^{-1}.

Synthesis of **2**

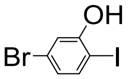

KI (3.32 g, 20 mmol) and KIO_3 (1.86 g, 8.7 mmol) were added to a stirred suspension of 3-bromophenol (5.00 g, 28.9 mmol) in a mixed solvent of H_2O (120 mL) and H_2SO_4 (3 mL), and the mixture was stirred at r.t. After 18 h, the reaction was quenched with $Na_2S_2O_4$ aq. and filtrated with Celite. The solution was extracted with EtOAc, and the organic layer was washed with brine and $NaHCO_3$ aq., dried over Na_2SO_4. The solvent was removed under reduced pressure, and the residue was purified by column chromatography on silica gel (hexane) and preparative SEC using $CHCl_3$ as the eluent, to give **2** as a white solid (4.08 g, 47%). ^{1}H NMR (400 MHz, $CDCl_3$, r.t.) : δ_H = 7.47 (d, *J* = 8.2 Hz, 1H, *m*-Ph), 7.14 (d, *J* = 2.3 Hz, 1H, *o*-Ph), 6.82 (dd, J_1 = 8.4 Hz, J_2 = 2.0 Hz, 1H, *p*-Ph), 5.34 (s, 1H, -OH); ^{13}C NMR (100 MHz, $CDCl_3$, r.t.): δ_c = 155.52, 138.89, 125.60, 123.43, 118.39, 83.95; Anal. Calcd for C_6H_4BrIO: C, 24.11; H, 1.35; O, 5.35; Br, 26.73%; Found: C, 24.19; H, 1.38; O, 5.42; Br, 26.51%.

Synthesis of **3**

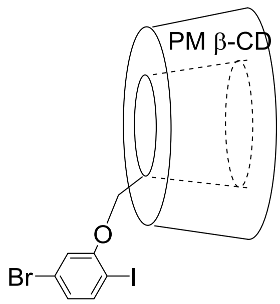

A suspension of **2** (328 mg, 1.1 mmol), 6-*O*-monotosyl β-cyclodextrin permethylate (1.57 g, 1.0 mmol), and dry K_2CO_3 (691 mg, 5.0 mmol) in DMF (15 mL) was stirred at 100 °C for 24 h. The mixture was filtrated with Celite and extracted with EtOAc and washed with brine. The organic layer was separated and dried over Na_2SO_4. The solvent was removed under reduced pressure to give **3** as a white solid (1.63 g, 96%). MALDI-TOF MS (CHCA): *m/z* 1719 ([**3**+Na]$^+$, $C_{68}H_{112}BrINaO_{35}$, calcd 1718); ^{1}H NMR (400 MHz, CD_2Cl_2, r.t.) : δ_H = 7.62 (d, J = 8.2 Hz, 1H, *m*-Ph), 6.99 (d, J = 2.3 Hz, 1H, *o*-Ph), 6.89 (dd, J_1 = 8.4 Hz, J_2 = 2.0 Hz, 1H, *p*-Ph), 5.32-4.75 (m, 9H, CD-H_1), 4.12-3.05 (m, 100H, CD-H_2, OCH_3); ^{13}C NMR(100 MHz, CD_2Cl_2, r.t.) : δ_C = 171.15, 158.29, 140.42, 125.89, 123.07, 116.01, 99.30, 99.14, 99.07, 98.96, 98.94, 98.89, 84.73, 82.54, 82.50, 82.48, 82.45-82.37 (several peaks overlapped), 82.32, 82.28, 82.21, 82.17, 81.46, 80.55, 80.43, 80.40, 80.13, 80.07 (several peaks overlapped), 72.45, 72.07, 72.04, 71.95, 71.79, 71.45, 71.42, 71.36, 71.34, 71.61, 69.11, 61.89, 61.59, 61.55, 61.53, 61.45, 60.58, 59.29, 59.14 (several peaks overlapped), 59.07, 59.02, 58.99, 58.94 (several peaks overlapped), 58.90, 58.80, 58.65, 58.60, 58.25.; Anal. Calcd for $C_{68}H_{112}BrIO_{35}$: C, 48.14; H, 6.65; O, 33.01; Br, 4.71; I, 7.48%; Found: C, 47.89; H, 6.72; O, 32.84; Br, 4.70; I, 7.23%.

Synthesis of **4**

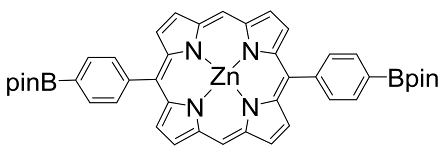

A solution of dipyrromethane (580 mg, 2.5 mmol) and 4-(4,4,5,5-tetramethyl-1,3,2-dioxaborolan- 2-yl)benzaldehyde (365 mg, 2.5 mmol) in CH_2Cl_2 (850 mL) was stirred for 2 h, under argon, in the presence of $BF_3 \cdot OEt_2$ (210 μL). The reaction was quenched by the addition of DDQ (851 mg, 3.75 mmol) and stirring continued for 15 min. The mixture was basified with Et_3N (3 mL) and removed under reduced pressure. A suspension of The residue and $Zn(OAc)_2$ (2.3 g, 12.5 mmol) in CH_2Cl_2 (500 mL) was refluxed for 15 h. The mixture was diluted with CH_2Cl_2 and washed with brine. The organic layer was separated and dried over Na_2SO_4. The solvent was removed under reduced pressure and the resulting residue was recrystallized from CH_2Cl_2 / MeOH to give **4** as a purple crystals (788 mg, 41%). MALDI-TOF MS (DIT): *m/z* 775.8 ($[M]^+$, $C_{44}H_{42}B_2N_4O_4Zn$, calcd 776.3); ^{1}H NMR(400 MHz, $CDCl_3$-pyridine-d_5, r.t.) : δ_H = 10.21 (s, 2H, *meso*-H), 9.36 (d, *J* = 4.5 Hz, 4H, *β*-H), 9.06 (d, *J* = 4.5 Hz, 4H, *β*-H), 8.27 (d, *J* = 8.3 Hz, 4H, Ph-H_1), 8.24 (d, *J* = 4.5 Hz, 4H, Ph-H_2), 1.51 (s, 24H, pinacol-H); ^{13}C NMR (100 MHz, $CDCl_3$-pyridine-d_5, r.t.): δ_c = 149.74, 149.41, 146.37, 134.40, 132.82, 132.09, 131.52, 119.35, 105.81, 84.03, 67.91, 25.06.

Synthesis of **5**

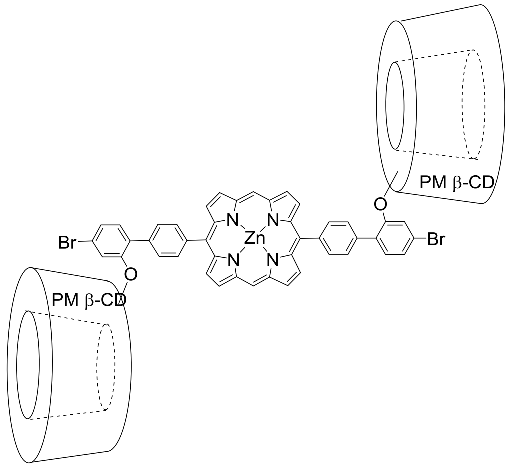

A solution of **3** (326 mg, 0.19 mmol), **4** (62 mg, 0.08 mmol), Na_2CO_3 (61 mg, 0.58 mmol) and $Pd(PPh_3)_4$ (9.2 mg, 0.0082 mmol) in a mixed solvent of toluene (5 mL), EtOH (1 mL) and H_2O (2 mL) was stirred at 70 °C for 40 h. The mixture was extracted with EtOAc and washed with brine. The organic layer was separated and dried over Na_2SO_4. The solvent was removed under reduced pressure, and the residue was purified by preparative SEC using $CHCl_3$ as the eluent, to give **5** as a purple solid (213 mg, 72%). MALDI-TOF MS (CHCA): *m/z* 3683.4 ($[M+Na]^+$, $C_{168}H_{242}Br_2N_4NaO_{70}Zn$, calcd 3683.3); ^1H NMR (400 MHz, CD_2Cl_2, r.t.) : δ_H = 10.41 (s, 2H, *meso*-H), 9.54 (d, *J* = 4.5 Hz, 4H, *β*-H), 9.32 (d, *J* = 4.5 Hz, 4H, *β*-H), 8.34 (d, *J* = 7.6 Hz, 4H, Ph-H_1), 8.02 (d, *J* = 7.6 Hz, 4H, Ph-H_2), 7.64 (d, *J* = 7.6 Hz, 2H, Ph-H_3), 7.39 (dd, J_1 = 7.9 Hz, J_2 = 1.9 Hz, 2H, Ph-H_4), 7.34 (d, *J* = 1.5 Hz, 2H, Ph-H_5), 5.26-4.97 (m, 16H, CD-H_1), 4.28-3.03 (m, 202H, CD-H_2, OCH_3); ^{13}C NMR (100 MHz, $CDCl_3$, r.t.): δ_c = 156.98, 150.56, 149.95, 142.05, 136.92, 134.87, 133.52, 133.23, 132.40, 130.14, 128.31, 124.55, 122.19, 120.18, 116.39, 106.57, 99.34, 99.24, 99.17, 98.99, 98.93, 82.70, 82.57, 82.52, 82.48, 82.45, 82.39, 82.32, 82.25, 82.16, 81.54, 80.52, 80.39, 80.22, 80.15, 79.98, 72.46, 72.15, 71.97, 71.88, 71.46, 71.42, 71.33, 70.83, 62.05, 61.59, 91.57, 61.54, 61.48, 61.45, 59.35, 59.29, 59.12, 59.09, 58.85, 58.79, 58.75, 58.68, 58.66, 58.13.; Anal. Calcd for

$C_{168}H_{242}Br_2N_4O_{70}Zn$ ▪ $7H_2O$: C, 53.25; H, 6.81; Br, 4.22;N, 1.48; O, 32.51; Zn, 1.73%; Found: C, 53.00; H, 6.54; N, 1.23%.

Synthesis of **7**

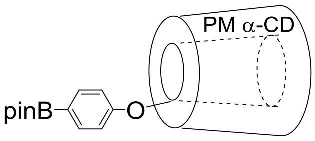

A suspension of *p*-iodophenol (242 mg, 1.1 mmol), 6-*O*-monotosyl α-cyclodextrin permethylate (1.37 g, 1.0 mmol), and dry K_2CO_3 (691 mg, 5.0 mmol) in DMF (15 mL) was stirred at 100 °C for 20 h. The mixture was extracted with EtOAc and washed with brine. The organic layer was separated and dried over Na_2SO_4. The solvent was removed under reduced pressure to give **7'** as a white solid (1.45 g, 99%). A suspension of **7'** (212 mg, 0.15 mmol), B_2pin_2 (76.2 mg 0.3 mmol), KOAc (44 mg, 0.45 mmol) and $PdCl_2$(dppf) (12.3 mg, 0.015 mmol) in DMSO (3 mL) was stirred at 70 °C for 24 h. The mixture was extracted with EtOAc and washed with brine. The organic layer was separated and dried over Na_2SO_4. The solvent was removed under reduced pressure, and the residue was purified by column chromatography on silica gel (EtOAc:EtOH = 99:1), to give **7** as a white solid (152 mg, 72%). MALDI-TOF MS(CHCA): *m/z* 1436.5 ($[M+Na]^+$, $C_{65}H_{109}BNaO_{32}$, calcd 1435.7); ^{1}H NMR (400 MHz, $CDCl_3$, r.t.) : δ_H = 7.74 (d, *J* = 8.6 Hz, 2H, Ph-H_1), 6.93 (d, *J* = 8.6 Hz, 2H, Ph-H_2), 5.10-4.99 (m, 6H, CD-H_1), 4.38-3.13 (m, 87H, CD-H_2, OCH_3), 1.32 (s, 12H, pinacol-H); ^{13}C NMR (100 MHz, $CDCl_3$, r.t.): δ_c = 161.36, 136.49, 113.92, 100.21, 100.19, 100.08, 100.05, 100.01, 99.77, 83.57, 82.86, 82.83, 82.54, 82.37, 82.29, 82.28, 82.24, 82.20, 82.12, 82.08, 82.01, 81.40, 81.33, 81.29, 71.55, 71.34 (several peaks overlapped), 71.25, 71.21,

71.19 (several peaks overlapped), 71.09, 70.77, 67.67, 61.89, 61.82, 61.78, 60.38, 59.05, 59.00, 58.95, 58.92, 58.06, 57.92, 57.87, 57.82, 24.85, 24.83, 24.61, 21.05, 14.20.

Synthesis of **8**

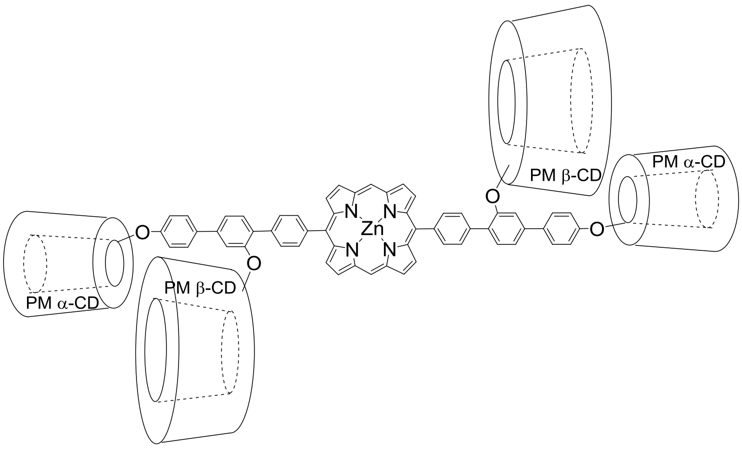

A suspension of **5** (9.2 mg, 2.5 μmol), **7** (14.1 mg, 10 μmol), Cs_2CO_3 (4.9 mg, 15 μmol) and $Pd(PPh_3)_4$ (0.29 mg, 0.25 μmol) in a mixed solvent of toluene (5 mL) and H_2O (1 mL) was stirred at 70 °C for 50 h. The mixture was extracted with EtOAc and washed with brine. The organic layer was separated and dried over Na_2SO_4. The solvent was removed under reduced pressure, and the residue was purified by preparative SEC using $CHCl_3$ as the eluent, to give **8** as a purple solid (6 mg, 40%). MALDI-TOF MS (CHCA): *m/z* 6096.2 ($[M+Na]^+$, $C_{286}H_{436}N_4NaO_{130}Zn$, calcd 6096.7); ^{1}H NMR(400 MHz, CD_2Cl_2, r.t.) : δ_H = 10.43 (s, 2H, *meso*-H), 9.56 (d, *J* = 4.5 Hz, 4H, *β*-H), 9.38 (d, *J* = 4.5 Hz, 4H, *β*-H), 8.35 (d, *J* = 8.3 Hz, 4H, Ph-H_1), 8.10 (d, *J* = 7.6 Hz, 4H, Ph-H_2), 7.81 (d, *J* =7.6 Hz, 2H, Ph-H_3), 7.69 (d, *J* = 8.3 Hz, 4H, Ph-H_4), 7.42 (d, *J* = 8.3 Hz, 2H, Ph-H_5), 7.36 (s, 2H, Ph-H_6), 7.13 (d, *J* = 8.3 Hz, 4H, Ph-H_7), 5.54-4.97 (m, 36H, CD-H_1), 4.60-2.95 (m, 368H, CD-H_2, OCH_3).

Synthesis of **9**

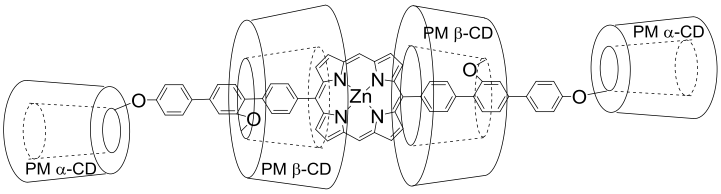

A suspension of **5** (9.2 mg, 2.5 μmol) in a mixed solvent of MeOH (2 mL) and H_2O (2 mL) was stirred at 50 °C. After 24 h, **7** (14.1 mg, 10 μmol), Cs_2CO_3 (4.9 mg, 15 μmol), $Pd(OAc)_2$ (0.06 mg, 0.25 μmol) and TXPTS (0.33 mg, 0.5 μmol) were added to the solution and stirred at 70 °C for 24 h. The mixture was extracted with EtOAc and washed with brine. The organic layer was separated and dried over Na_2SO_4. The solvent was removed under reduced pressure, and the residue was purified by preparative SEC using $CHCl_3$ as the eluent, to give **9** as a purple solid (5 mg, 31%). MALDI-TOF MS (CHCA): *m/z* 6097.6 ($[M+Na]^+$, $C_{286}H_{436}N_4NaO_{130}Zn$, calcd 6096.7); ^{1}H NMR(400 MHz, CD_2Cl_2, r.t.) : δ_H = 10.03 (s, 2H, *meso*-H), 9.53 (d, *J* = 3.8 Hz, 4H, *β*-H), 9.34 (d, *J* = 3.8 Hz, 4H, *β*-H), 8.62 (d, *J* = 7.6 Hz, 4H, Ph-H_1), 8.00 (d, *J* = 8.3 Hz, 2H, Ph-H_2), 7.95 (d, *J* = 7.6 Hz, 4H, Ph-H_3), 7.72 (d, *J* = 8.3 Hz, 4H, Ph-H_4), 7.63-7.61(m, 4H, Ph-H_5,H_6), 7.13 (d, *J* = 8.3 Hz, 4H, Ph-H_7), 5.54-4.95 (m, 36H, CD-H_1), 4.71-2.69 (m, 368H, CD-H_2, OCH_3).

Synthesis of **12**

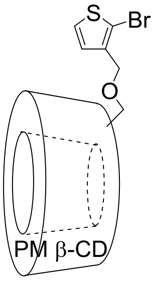

A dried flask was charged with 6-*O*-monohydroxy permethylated β-cyclodextrin (2.4 g, 1.6 mmol), THF (30 mL), **11** (0.75 g, 3.0 mmol) in THF (30 mL) and NaH (60% oil dispersion, 1.2 g, 29 mmol) and the mixture was stirred at 60 °C overnight. The reaction was quenched with H_2O, the mixture was extracted with EtOAc and washed with brine. The organic layer was dried over Na_2SO_4 and evaporated in vacuo. The residue was purified by column chromatography (silica-gel; EtOAc / EtOH = 99 / 1) to give **12** (1.3 g, 51%). MALDI-TOF MS (CHCA) : *m/z* 1611.58 ([**12**+Na]$^+$, $C_{67}H_{113}BrO_{35}S$, calcd. 1588.60); ^{1}H NMR (400 MHz, CD_2Cl_2, r.t.) : δ_H = 7.27 (d, J = 4.5 Hz, 1H), 7.00 (d, J = 4.5 Hz, 1H), 5.10-5.08 (m, 8H), 3.81-3.11 (m, 114H).; ^{13}C NMR (126 MHz, $CDCl_3$, r.t.) : δ_c = 138.31, 128.26, 125.82, 110.64, 99.01, 98.93 (several peaks overlapped), 98.88, 82.07-82.03 (several peaks overlapped), 81.95, 81.84-81.75 (several peaks overlapped), 80.56, 80.34, 80.22, 80.12, 71.42 (several peaks overlapped), 71.28, 71.03, 70.94-70.90 (several peaks overlapped), 69.36, 67.36, 61.49-61.40 (several peaks overlapped), 58.97-58.95 (several peaks overlapped), 58.54-58.47 (several peaks overlapped).

Synthesis of ([2,2'-bithiophen]-5-ylethynyl)triisopropylsilane

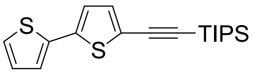

A dried flask was charged 5-bromo-2,2'-bithiophene (2.06 g, 8.4 mmol), $PdCl_2(PPh_3)_2$ (0.593 g, 0.85 mmol), CuI (32.3 mg, 0.17 mmol) and Et_3N (40 mL). (triisopropylsilyl)acetylene (2.8 mL, 12 mmol) was added and the mixture was refluxed overnight. NH_4Cl was added and filtered through a pad of Celite. The mixture was poured into 1N HCl aq., extracted with EtOAc and washed with brine. The organic layer was dried over Na_2SO_4 and evaporated in vacuo. The residue was purified by preparative GPC to give ([2,2'-bithiophen]-5-ylethynyl)triisopropylsilane (1.47 g, 54%). ^{1}H NMR (500 MHz, $CDCl_3$, r.t.) : δ_H = 7.13 (dd, J

= 1.1, 5.1 Hz, 1H), 7.10 (dd, J = 1.1, 3.7 Hz, 1H), 7.07 (d, J = 3.8 Hz, 1H), 6.93 (d, J = 3.8 Hz, 1H), 6.92 (d, J = 3.7 Hz, 1H), 1.12 (s, 21H).; ^{13}C NMR (126 MHz, $CDCl_3$, r.t.): δ_c = 138.71, 136.77, 133.25, 127.97, 124.99, 124.21, 123.26, 122.28, 99.45, 96.68, 18.78, 11.45.

Synthesis of **13**

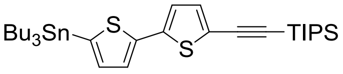

A dried flask was charged with ([2,2'-bithiophen]-5-ylethynyl)triisopropylsilane (0.90 g, 2.8 mmol) and THF (10 mL). *n*BuLi (1.7 mL of 1.65 M solution in hexane, 2.8 mmol) was added dropwise at -78 °C and the mixture was stirred at -78 °C for 1 h. *n*Bu_3SnCl (0.91 mL, 3.4 mmol) was added dropwise at room temperature and the mixture was stirred at room temperature overnight. The mixture was poured into 1N HCl aq. and extracted with CH_2Cl_2. The organic layer was dried over Na_2SO_4 and evaporated in vacuo. The residue was purified by column chromatography (10% w/w anhydrous K_2CO_3 / silica-gel; CH_2Cl_2) and preparative GPC to give **13** (1.1 g, 62%). ^{1}H NMR (500 MHz, $CDCl_3$, r.t.) : δ_H = 7.27 (d, J = 3.3 Hz, 1H), 7.10 (d, J = 3.8 Hz, 1H), 7.05 (d, J = 3.4 Hz, 1H), 6.99 (d, J = 3.8 Hz, 1H), 1.58 (m, 6H), 1.35 (m, 6H), 1.12 (m, 27H), 0.90 (m, 9H).; ^{13}C NMR(126 MHz, $CDCl_3$, r.t.) : δ_c = 142.08, 138.94, 137.65, 136.16, 133.21, 125.32, 122.92, 121.76, 99.48, 96.41, 28.94, 27.26, 18.67, 13.66, 11.34, 10.91.

Synthesis of **14**

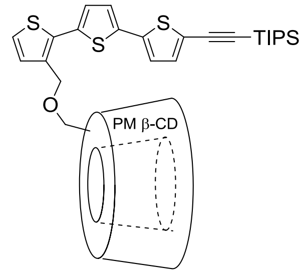

A dried flask was charged with **13** (0.321 g, 0.51 mmol) in CH_2Cl_2 and the solvent was removed under reduced pressure. **12** (0.536 g, 0.34 mmol), $Pd(PPh_3)_4$ (0.039 g, 0.034 mmol) and toluene (25 mL) were added and the mixture was refluxed overnight. The mixture was purified by column chromatography (10% w/w anhydrous K_2CO_3 / silica-gel; EtOAc / toluene = 1 / 1, EtOAc / EtOH = 95 / 5) to give **14** (0.433 g, 69%). ^1H NMR (400 MHz, CD_2Cl_2, r.t.) : δ_H = 7.16 (d, *J* = 5.4 Hz, 1H), 7.08-7.04 (m, 4H), 6.99 (d, *J* = 4.1 Hz, 1H), 5.03-4.99 (m, 7H), 3.77-3.00 (m, 104H).

Synthesis of **17**

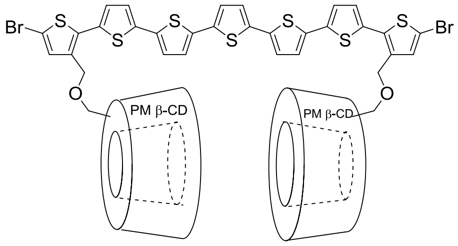

14 (1.04 g, 0.56 mmol) was dissolved in THF (50 mL) and TBAF (2.8 mL of 1.0 M solution in THF, 2.8 mmol) was added. The solution was stirred at room temperature for 1 h and the solvent was removed under reduced pressure. The residue was purified by preparative GPC to give **15** (0.916 g, 96%). **15** (0.837 g, 0.49 mmol) and CuCl (15.1 mg, 0.15 mmol) were dissolved pyridine (15 mL) and the mixture was stirred at 40 °C overnight. The mixture was

poured into 1N HCl aq. and extracted with $CHCl_3$. The organic layer was dried over Na_2SO_4 and evaporated in vacuo. The residue was purified by preparative GPC to give **16** (0.594 g, 71%). Under argon atmosphere, **16** (0.490 g, 0.14 mmol), Na_2S (96.7 mg, 1.2 mmol) and KOH (19.3 mg, 0.34 mmol) were dissolved in 1,4-dioxane and the mixture was refluxed overnight. The mixture was poured into 1N HCl aq. and extracted with $CHCl_3$. The organic layer was dried over Na_2SO_4 and evaporated in vacuo. The residue was purified by preparative GPC to give heptathiophene with two PMCDs (0.320 g, 65%). Under argon atmosphere, thus formed heptathiophene (85 mg, 25 μmol) was dissolved in $CHCl_3$ (3.0 mL) and glacial AcOH (3.0 mL). To the solution, NBS (9.2 mg, 52 μmol) was added portionwisely at 0 °C and the mixture was stirred at 0 °C for 2 h. The mixture was poured into sat. $NaHCO_3$ aq. and extracted with $CHCl_3$. The organic layer was dried over Na_2SO_4 and evaporated in vacuo. The residue was purified by preparative GPC to give **17** (78 mg, 88%). ^{1}H NMR (400 MHz, CD_2Cl_2, r.t.) : δ_H = 7.09-7.02 (m, 12H), 5.06-4.95 (m, 16H), 4.53-4.52 (m, 4H), 4.07-3.02 (m, 262H).

Synthesis of 1,8-diaminonaphthalene protected *p*-bromobenzeneboronic acid

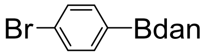

A suspension of *p*-bromobenzeneboronic acid (4.02 g, 20 mmol) and 1,8-diaminonaphthalene (dan) (3.48 g, 22 mmol) in toluene (200 mL) was refluxed for 3 h. The solvent was removed under reduced pressure, and the residue was purified by column chromatography on silica gel (Hexane : EtOAc = 10 : 1), to give 1,8-diaminonaphthalene protected *p*-bromobenzeneboronic acid as a white solid (6.91 mg, 99%). MALDI-TOF MS (DIT): *m/z* 321.9 ($C_{16}H_{12}BBrN_2$, calcd 322.0); ^{1}H NMR (400 MHz, $CDCl_3$, r.t.) : δ_H = 7.56 (d, *J* = 8.2

Hz, 2H, Ph-H_1), 7.44 (d, *J* =8.2 Hz, 2H, Ph-H_2), 7.17 (t, *J* = 7.7 Hz, 2H, *m*-dan), 7.10 (d, *J* = 7.2 Hz, 2H, *o*-dan), 6.40 (dd, J_1 = 7.2 Hz, J_2 = 0.9 Hz, 2H, *p*-dan), 5.92 (br, 2H, N-H); ^{13}C NMR (100 MHz, $CDCl_3$, r.t.): δ_c = 140.70, 136.24, 132.92, 131.31, 127.56, 124.80, 119.76, 117.97, 106.12.

Synthesis of **18**

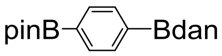

A suspension of 1,8-diaminonaphthalene protected *p*-bromobenzeneboronic acid (1.61 g, 5.0 mmol), B_2pin_2 (1.90 g 7.5 mmol), KOAc (1.47 g, 15.0 mmol) and $PdCl_2$(dppf) (204 mg, 0.25 mmol) in DMF (100 mL) was stirred at 80 °C for 3 h. The mixture was extracted with EtOAc and washed with brine. The organic layer was separated and dried over Na_2SO_4. The solvent was removed under reduced pressure, and the residue was purified by column chromatography on silica gel (EtOAc), preparative SEC using $CHCl_3$ as the eluent, and recrystallization (CH_2Cl_2-Hexane) to give **18** as a white solid (1.34 g, 72%). MALDI-TOF MS (DIT): *m/z* 369.9 ([**18**]$^+$, $C_{22}H_{24}B_2N_2O_2$, calcd 370.2); ^{1}H NMR (400 MHz, $CDCl_3$, r.t.) : δ_H = 7.93 (d, *J* =8.2 Hz, 2H, Ph-H_1), 7.69 (d, *J* =8.2 Hz, 2H, Ph-H_2), 7.18 (t, *J* =7.7 Hz, 2H, Naphthalene-H_1), 7.10 (d, *J* =7.7 Hz, 2H, Naphthalene-H_2), 6.45 (d, *J* =7.7 Hz, 2H Naphthalene-H_2), 6.09 (s, 2H, N-H), 1.42 (s, 12H, pinacol-H); ^{13}C NMR (100 MHz, $CDCl_3$, r.t.): δ_c = 140.99, 136.32, 134.41, 130.67, 127.59, 119.90, 117.82, 106.05, 83.92, 24.87.

Synthesis of **19**

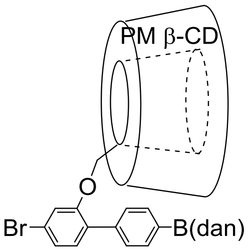

A solution of **3** (597 mg, 0.35 mmol), **18** (143 mg, 0.39 mmol), Na_2CO_3 (112 mg, 1.06 mmol) and $Pd(PPh_3)_4$ (20.3 mg, 0.018 mmol) in a mixed solvent of toluene (5 mL), EtOH (1 mL) and H_2O (2 mL) was stirred at 70 °C for 14 h. The mixture was extracted with EtOAc and washed with brine. The organic layer was separated and dried over Na_2SO_4. The solvent was removed under reduced pressure, and the residue was purified by column chromatography on silica gel (EtOAc:EtOH = 99:1), to give **19** as a white solid (555 mg, 87%). MALDI-TOF MS (CHCA): *m/z* 1832.2 ([**19**+Na]$^+$, $C_{84}H_{124}BBrN_2NaO_{35}$, calcd 1833.7); ^{1}H NMR (400 MHz, CD_2Cl_2, r.t.) : δ_H = 7.71 (d, *J* = 8.2 Hz, 2H, Ph-H_1), 7.62 (d, *J* = 8.2 Hz, 2H, Ph-H_2), 7.29 (d, *J* = 7.7 Hz, 1H, Ph-H_3), 7.23-7.20 (m, 2H, Ph-$H_{4,}$ H_5), 7.15 (t, *J* = 7.7 Hz, 2H, *m*-dan), 7.04 (d, *J* = 8.2 Hz, 2H, *o*-dan), 6.49 (d, *J* = 7.2 Hz, 2H, *p*-dan), 6.38 (br, 2H, N-H) 5.32-4.63 (m, 9H, CD-H_1), 4.20-2.96 (m, 100H, CD-H_2, OCH_3).; ^{13}C NMR (126 MHz, $CDCl_3$, r.t.) : δ_c = 156.46, 141.19, 139.12, 136.39, 132.95, 132.14, 132.12, 132.06, 131.95, 131.92, 131.36, 131.14, 129.93, 129.51, 128.55, 128.45, 127.65, 124.25, 121.85, 119.92, 117.82, 116.34, 106.02, 99.00 (several peaks overlapped), 98.96, 98.92, 98.74, 82.11, 82.08, 82.06, 82.00, 81.93, 81.91, 81.81, 81.80, 81.77 (several peaks overlapped), 81.72, 81.67, 81.61, 80.72, 80.58, 80.41, 80.23 (several peaks overlapped), 79.71, 75.01, 71.69, 71.62, 71.46 (several peaks overlapped), 71.37, 71.04, 71.02, 70.99, 70.93 (several peaks overlapped), 70.88, 70.43, 68.07, 61.79, 61.50, 61.47, 61.41 (several peaks overlapped), 61.36, 60.39, 59.10, 59.05

(several peaks overlapped), 59.02 (several peaks overlapped), 58.98, 58.92, 58.69, 58.61, 58.56, 58.53, 58.46 (several peaks overlapped), 57.97.

Synthesis of **20**

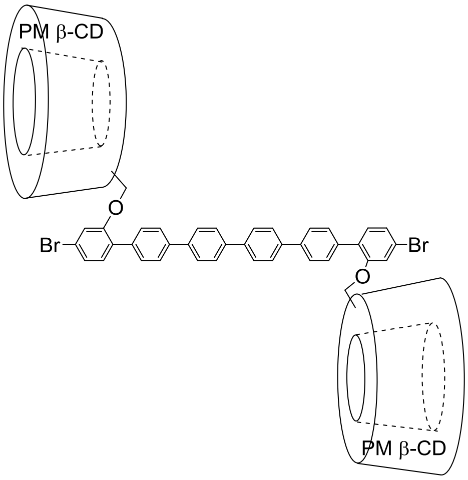

19 (86 mg, 0.047 mmol) was dissolved in THF (2 mL), and 5N HCl aq. (0.2 mL) was added. After 7 h, the mixture was extracted with EtOAc and washed with brine. The organic layer was separated and dried over Na_2SO_4. The solvent was removed under reduced pressure, to give dan deprotected boronic acid as a white solid (80 mg, 99%). A solution of thus formed deprotected boronic acid (162 mg, 0.096 mmol), 4,4'-diiodobiphenyl (16.24 mg, 0.04 mmol), Cs_2CO_3 (39.1 mg, 0.12 mmol) and $Pd(PPh_3)_4$ (4.6 mg, 0.004 mmol) in a mixed solvent of toluene (2.5 mL), EtOH (0.5 mL) and H_2O (1.0 mL) was stirred at 70 °C for 40 h. The mixture was extracted with EtOAc and washed with brine. The organic layer was separated and dried over Na_2SO_4. The solvent was removed under reduced pressure, and the residue was purified by preparative SEC using $CHCl_3$ as the eluent, to give **20** as a white solid (85 mg, 62%). MALDI-TOF MS(CHCA): *m/z* 3466.0 ($[M+Na]^+$, $C_{160}H_{240}Br_2NaO_{70}$, calcd 3465.3); ^1H NMR(400 MHz, CD_2Cl_2, r.t.) : δ_H = 7.78 (dd, J_1 =14.5 Hz, J_2 =8.6 Hz, 8H, Ph-H_1), 7.70 (dd, J_1 =13.1 Hz, J_2 =8.6 Hz, 8H, Ph-H_2), 7.32-7.21 (m, 6H, Ph-H_3, H_4, H_5), 5.32-4.71 (m, 18H,

CD-H_1), 4.18-2.96 (m, 200H, CD-H_2, OCH_3); ^{13}C NMR (100 MHz, CD_2Cl_2, r.t.): δ_c = 156.83, 140.09, 139.91, 139.89, 136.86, 131.93, 130.48, 129.77, 127.83, 127.67, 126.87, 124.45, 121.97, 116.49, 99.15, 99.04, 98.96, 98.91 (several peaks overlapped), 82.55, 82.50, 82.47, 82.42, 82.38(several peaks overlapped), 82.22, 82.17, 82.13 (several peaks overlapped), 81.19, 80.52, 80.37, 80.34, 80.13 (several peaks overlapped), 79.82, 72.31, 72.16, 71.95, 71.85 (several peaks overlapped), 71.42, 71.37, 71.33, 70.63, 61.67, 61.56, 61.50, 61.47, 61.43, 59.20, 59.16, 59.10, 59.08, 59.05 (several peaks overlapped), 59.03, 58.81, 58.78, 58.74 (several peaks overlapped), 58.65, 57.76.; Anal. Calcd for $C_{160}H_{240}Br_2O_{70}\cdot 3H_2O$: C, 54.95; H, 7.09; Br, 4.57; O, 33.39%; Found: C, 54.70; H, 6.89%.

Synthesis of **23**

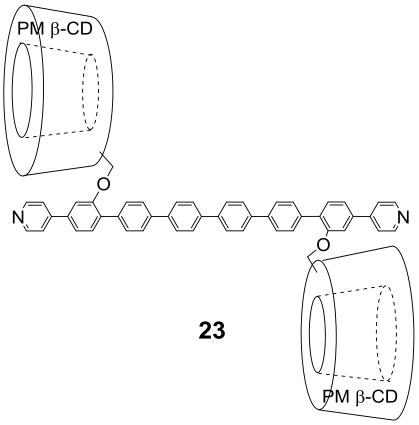

23

K_3PO_4 (19 mg, 0.088 mmol) was placed in a test tube and dried under reduced pressure with heating. The test tube was backfilled with argon. 4-pyridylboronic acid (72 mg, 0.59 mmol), $Pd_2(dba)_3$ (5.9 mg, 0.0064 mmol), XPhos (12 mg, 0.024 mmol) and 1-butanol (1.0 mL) were added. To the solution, **20** was added and the mixture was stirred at 100 °C overnight. The mixture was filtered through a pad of Celite and extracted with EtOAc. The organic layer was dried over Na_2SO_4 and the solvent was removed under reduced pressure. The crude product was purified by preparative GPC to give **23** as a white solid (61.6 mg, 60%). MALDI-TOF

MS (CHCA) : *m/z* 3439.60 ([**23**+H^+], $C_{170}H_{248}N_2O_{70}$, calcd. 3439.60); ^{1}H NMR (400 MHz, CD_2Cl_2, r.t.) : δ_H = 8.60 (br s, 4H), 7.72 (d, J = 3.2 Hz, 8H), 7.68 (d, J = 5.4 Hz, 8H), 7.54 (br s, 4H), 7.49 (d, J = 7.7 Hz, 2H), 7.32 (d, J = 7.7 Hz, 2H), 7.27 (s, 2H), 5.02-4.84 (m, 18H), 4.23-2.90 (m, 200H).; ^{13}C NMR (126 MHz, $CDCl_3$, r.t.) : δ_C = 156.44, 150.26, 148.27, 139.85, 139.82, 139.68, 139.61, 138.53, 136.74, 131.66, 131.40, 131.26, 130.22, 130.06, 129.55, 127.55, 127.39, 126.56, 124.25, 121.59, 119.94, 116.23, 111.49, 99.03-98.95 (several peaks overlapped), 98.46, 82.17, 82.08-81.97 (several peaks overlapped), 81.85-81.69 (several peaks overlapped), 81.61, 81.50, 80.72, 80.64, 80.58, 80.43, 80.33-80.26 (several peaks overlapped), 80.13, 79.84, 79.40, 71.71, 71.56-71.46 (several peaks overlapped), 71.08, 71.00-70.92 (several peaks overlapped), 70.84, 70.63, 67.73, 61.57, 61.50, 61.47, 61.43, 61.40, 61.35, 61.33, 59.14, 59.10, 59.03 (several peaks overlapped), 58.96, 58.79, 58.63, 58.56, 58.52, 58.50, 58.41, 57.69.

Synthesis of **26**

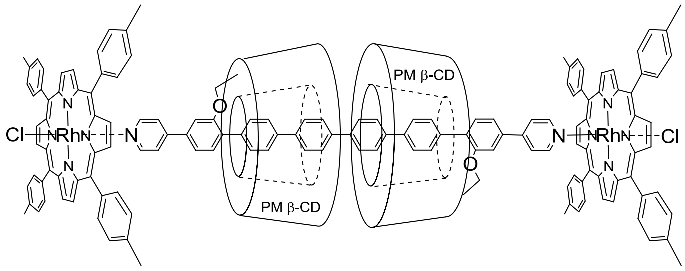

23 (61.6 mg, 18 μmol) was dissolved in MeOH (2.0 mL), H_2O (4.0 mL) and warmed at 70 °C for 3 h. The solvent was removed under reduced pressure and the residue was dissolved in $CHCl_3$ (5.0 mL). **25** (36.8 mg, 44 μmol) was added and the mixture was stirred at room temperature for 2 h. The mixture was purified by preparative GPC to give **26** (15.4 mg, 17%). ^{1}H NMR (400 MHz, CD_2Cl_2, r.t.) : δ_H = 8.84 (s, 16H), 8.09 (d, J = 7.3 Hz, 8H), 7.92 (d, J = 7.3 Hz, 8H), 7.88 (d, J = 8.8 Hz, 4H), 7.67 (d, J = 8.3 Hz, 4H), 7.51 (d, J = 7.3 Hz, 8H), 7.46

(d, J = 6.8 Hz, 8H), 7.44 (d, J = 7.8 Hz, 4H), 7.26 (d, J = 7.8 Hz, 4H), 6.99 (d, J = 8.3 Hz, 2H), 6.33 (d, J = 7.8 Hz, 2H), 6.27 (s, 2H), 5.27 (d, J = 6.8 Hz, 4H), 4.88-4.52 (m, 14H), 3.73-2.38 (m, 224H), 0.93 (d, J = 6.3 Hz, 4H); ^{13}C NMR (126 MHz, $CDCl_3$, r.t.) : δ_C = 155.81, 146.49, 145.81, 142.88, 142.61, 142.46, 139.71, 139.60, 139.49, 139.21, 137.21, 136.01, 134.75, 134.68, 134.61, 134.22, 134.04, 133.77, 132.43, 132.37, 132.28, 132.06, 129.89, 127.65, 127.56, 127.41, 127.21, 127.01, 126.37, 121.26, 121.17, 119.43, 99.13, 99.07, 98.95, 98.75, 98.64, 98.58, 98.04, 82.40, 82.22, 82.07-82.03 (several peaks overlapped), 81.88, 81.77-81.73 (several peaks overlapped), 81.67, 81.56, 81.44, 81.34, 81.17, 80.85, 80.74, 80.32, 80.01, 79.70, 78.31, 71.90, 71.77, 71.54, 71.46, 71.26, 71.18-71.14 (several peaks overlapped), 70.98, 70.89-70.85 (several peaks overlapped), 70.52, 70.45, 61.68, 61.60, 61.56, 61.46, 61.36, 61.15, 60.91, 59.39, 59.04 (several peaks overlapped), 59.00, 58.89, 58.86 (several peaks overlapped), 58.76, 58.39, 58.23, 58.18, 57.52, 53.42, 21.52.

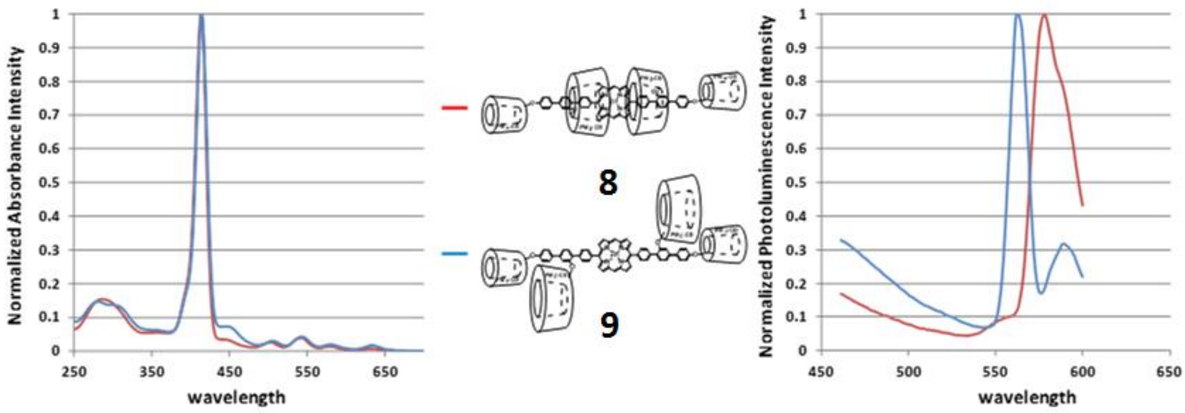

Figure S1: Absorption and emission spectra of **8** (red line) and **9** (blue line) in $CDCl_3$. The absorption spectra were normalised at the peak amplitude.

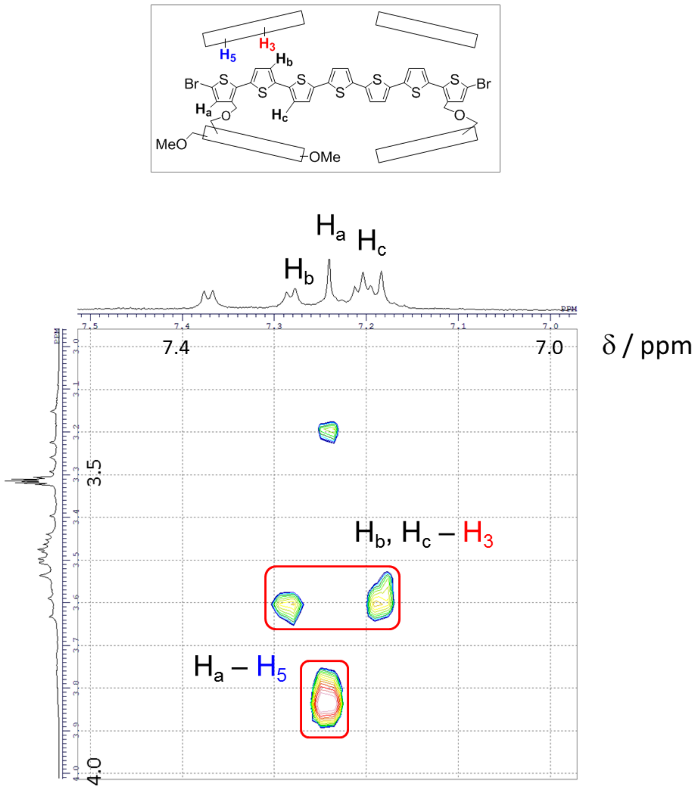

Figure S2: Partial ROESY NMR spectrum of **21** (400 MHz, $CDCl_3$) showing the NOEs between aromatic protons of the axial heptathiophene and inner protons of cyclodextrins.
